# Chest wall strapping in a porcine model: A model for the physiology of the oversized lung allograft

**DOI:** 10.1016/j.jhlto.2025.100248

**Published:** 2025-03-17

**Authors:** Eric Abston, Michael Eberlein

**Affiliations:** a Massachusetts General Hospital, Department of Thoracic Surgery, Boston, Massachusetts; b University of Wisconsin – Madison, Division of Allergy, Pulmonary and Critical Care, Madison, Wisconsin

**Keywords:** chest wall strapping, airway parenchymal interdependence, atelectasis, lung size mismatch, lung transplantation

## Abstract

Restrictive lung disease (RLD) is the leading indication for lung transplantation. Donor-to-recipient size matching is challenging in RLD, especially oversizing. Chest-Wall-Strapping (CWS) is a technique forcing the lung to operate at lower volumes. We developed a porcine model of CWS to investigate limits of oversizing. Farm-raised pigs were intubated and mechanically ventilated. Computer tomography (CT) volumetry demonstrated that Total Lung Volume (TLV) at 25 cm H_2_O end-expiratory pressure was 1,204 ml at baseline. Application of increasing doses of CWS reduced TLV to 879 ml (CWS-cuff inflated to 35 mm Hg) and reduced TLV to 620 ml (CWS-cuff inflated to 50 mm Hg). At 50 mm Hg of CWS (50% TLV reduction) atelectasis of the lung bases was evident. Lung elastance was increased with CWS. CT lung-density at 35 mm Hg CWS (30% reduction in TLV) was normal for the inflation state of the lung. Physiological limits of oversizing should be considered for optimal lung function.

Restrictive lung disease (RLD) is the leading indication for lung transplantation (LTx). Dono-to-recipient lung size matching is challenging in RLD. Often undersized allografts are preferred, as RLD is associated with smaller chest cavity size. LTx-candidates of short stature have lower transplant rates and higher risk of death while awaiting transplant.^1^ This disparity disproportionally disadvantages female candidates.^1^

In general oversizing in LTx can be associated with improved outcomes.^2^, ^3^ The physiology and the limits of “oversizing” are not well defined. Chest wall strapping (CWS) involves applying an external device to decrease chest wall compliance and reduce lung volumes. CWS can simulate aspects of oversized lungs transplanted into a smaller chest cavity.^4^, ^5^ In this investigation we describe a porcine model of CWS. The goal was to describe a simplified model system for the physiology of an “oversized” allograft and to investigate the limits of oversizing while maintaining a fully aerated lung.

## Methods

Farm raised pigs weighing 20–23 kg were anesthetized, paralyzed, intubated and mechanically ventilated. An esophageal balloon was placed. Animals then underwent CWS Protocol #1, followed by #2.

CWS Protocol #1: Dose-response protocol. Baseline Computer tomography (CT) scans and lung mechanics during respiratory pause (25 cm H_2_O) were obtained. A CWS (adult femoral blood pressure cuff) was placed circumferentially around the animal’s body, extending from immediately behind the forelegs distally to near the umbilicus. The CWS was inflated in three separate maneuvers to 20, 35, and 50 mm Hg.

CWS Protocol #2: CT scans and lung mechanics measurements were obtained stepwise during respiratory pauses with end-expiratory-pressure of 30, 25, 20, 15, 10, 5, 0 cm H_2_O unstrapped, followed by CWS to 35 mm Hg.

Chest wall geometry: CT scans were obtained at 30 and 0 cm H_2_O (to represent total-lung-capacity [TLC] and expiratory resreve volume) were evaluated in the coronal plane mid-way through the carina unstrapped, followed by CWS to 35 mm Hg.

All imaging was performed on a dual-source CT scanner (Somatom Deﬁnition Flash: Siemens). Image datasets were imported into Pulmonary Workstation 2.0 (VIDA Diagnostics) for post hoc analysis. Quantitative CT analysis was completed utilizing the Apollo Software (VIDA Diagnostics).

Statistics: Paired ANOVA tests were done using Prizm-GraphPad software. Linear best fit slopes were compared via paired *t*-test.

## Results

### CWS dose-response

**Figure 1:**
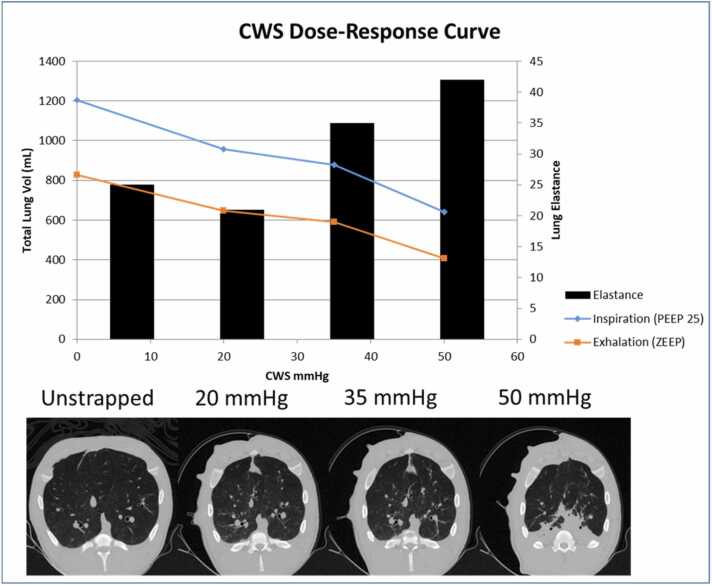
The effects of increased chest wall strapping (CWS) pressures on lung volumes and lung elastance. As pressure exerted on the chest wall increases via CWS, lung volumes diminish and lung elastance increases. Inspiratory CT scans show increasing chest strap pressure reduces thoracic size, and basilar atelectasis is seen at 50 mm Hg of CWS (N = 1). CT, computer tomography.

Total Lung Volume (TLV) at 25 cm H_2_O end-expiratory-pressure was 1,204 ml at baseline. Application of increasing doses of CWS reduced TLV to 879 ml at 35 mm Hg of CWS and to 620 ml at 50 mm Hg of CWS. Sagittal CT-images showed lung atelectasis at 50 mm Hg of CWS (50% reduction in TLV) (Figure 1). Dynamic lung parenchyma elastance was 25 cm H_2_O/liter at baseline and increased to 35 cm H_2_O/liter with of 35 mm Hg CWS and 42 cm H_2_O/liter with 50 mm Hg CWS.

### Thoracic geometry with CWS

**Figure 2:**
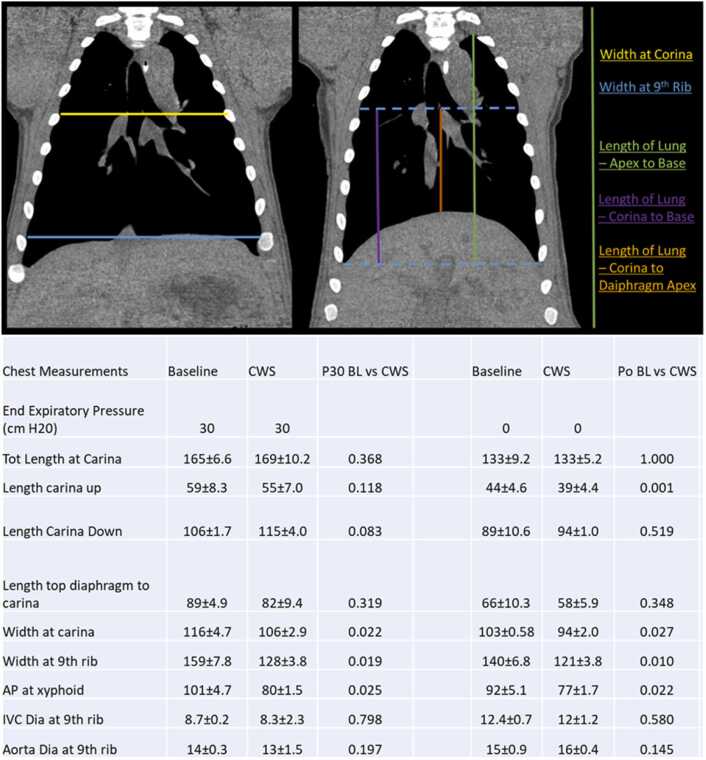
Thoracic geometry at baseline and CWS with maximal inflation (30 cm H_2_0) and exhalation (0 cm H_2_0). CWS significantly reduces width of the thoracic cage and AP diameter of the inflated at both inflation and exhalation. The total lung length is unchanged with CWS, but gas may be shifted distally as length from the carina up is diminished with CWS and trends toward being longer from the carina down (N = 3). CWS, chest wall strapping.

At 30 cm H_2_O (TLC), 35 mm Hg CWS significantly reduced width of the thorax at the carina (116 ± 4.7 mm vs 106 ± 2.9 mm, *p* = 0.020), width at the 9th rib (159 ± 7.8 mm vs 128 ± 3.8 mm, *p* = 0.018), and anteroposterior (AP) diameter at the xyphoid (101 ± 4.7 mm 80 ± 1.5 mm, *p* = 0.025). Length of the lung (Figure 2) was not significantly changed at inflation pressure of 30 cm H_2_O. Inferior vena cava and aortic diameter were not reduced by CWS at 30 and 0 cm H_2_O end-expiratory pressure. At 0 cm H_2_O end-expiratory pressure, the width of the thorax at the carina (103 ± 0.58 mm vs 94 ± 2.0 mm, *p* = 0.027), the 9th rib (140 ± 6.8 mm vs 121 ± 3.8 mm, *p* = 0.01), and the AP diameter (92 ± 5.1 mm vs 77 ± 1.7 mm, *p* = 0.022) were reduced. Additionally, carina-to-apex distance was reduced (44 ± 4.6 mm vs 39 ± 4.4 mm, *p* = 0.001) but the length of the lung was unchanged.

### CWS and parenchyma

**Figure 3:**
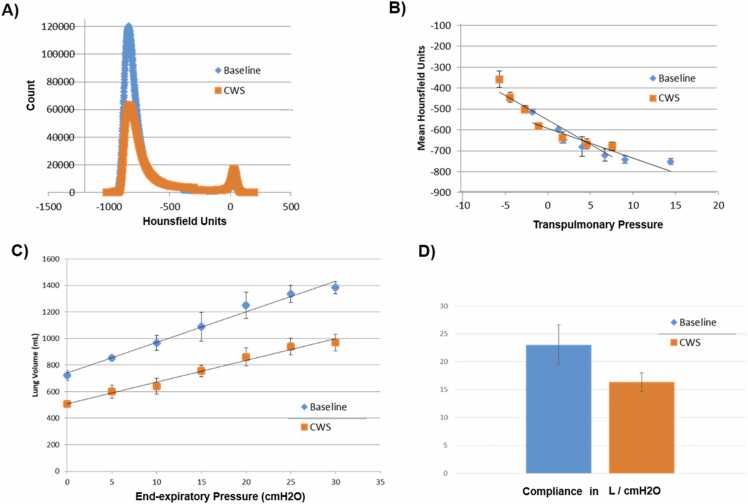
(A) Representative frequency-distribution plot showing same animal at baseline and with CWS (N = 1). (B) Mean Hounsfield units (Hu) for total lung through deflation. CWS significantly increases mean Hu *p* < 0.001. Interaction between CWS and deflation *p* = 0.039. Slope of best fit line *p* = 0.1 (*N* = 3). (C) CWS reduces Lung volumes by ANOVA *p* < 0.001 (N = 3). (D) Compliance is reduced by CWS (slope of linear best fit line from Figure 3C) *p* = 0.08 (*N* = 3). CWS, chest wall strapping.

CWS increased mean Hounsfield units averaged across the entire lung (*p* = 0.001, Figures 3A and B) across the deflation limb. The lung density curves over the deflation limbs of unstrapped and CWS have similar and overlapping slopes (Figure 3B).

### Lung mechanics during CWS

Total lung volumes were reduced with CWS at 35 mm Hg (*p* < 0.001; Figure 3C). The slope of lung volume at each inflation pressure versus represents static lung compliance and is reduced by CWS (*p* = 0.08, Figure 3D).

## Discussion

Here, we characterized the effects of CWS in a porcine model, quantifying the effects of CWS on thoracic geometry and lung parenchyma and lung mechanics as a model of an oversized lung transplant. CWS applies pressure to the thorax and upper abdomen and is capable of reducing thoracic width, lung volume, and compliance in a dose-dependent manner. Up to a reduction of 30% of TLV, the lung remains aerated with normal lung density, as predicted by the lung inflation state. However, a 50% reduction of TLV was associated with basilar lung atelectasis.

Donor-to-recipient lung size matching can be estimated by the predicted-total-lung-capacity-ratio (pTLCratio).^6^ There is a non-linear association between the pTLCratio and LTx survival: with higher pTLCratio from 0.5 to about 1.3 survival improves.^6^ At a pTLCratio of 1.3, an inflection occurs. When the pTLCratio exceeds 1.5 it is associated with lower survival. This inflection point of oversizing per pTLCratio seems to be in similar range, where we started to see CWS-related atelectasis in our porcine model.

In very severe RLD recipient-pTLC might not be a reliable metric of chest cavity size. At the time of the LTx-operation, the RLD-related small recipient chest cavity size increases intra- and perioperative complexity.^7^ The severity of RLD chest cavity size reduction might be estimated by actual-TLC, or CT-volumetry derived TLV of the recipient.^8^ Substantial oversizing on the basis of the CT-volumetric ratio was independently associated with an increased risk of death in LTx for severe ILD, whereas the pTLCratio did not have a significant association with survival.^8^ Experimental CWS data from this porcine model could provide general physiological information to guide maximal acceptable lung allograft sizes for the available chest cavity size of a recipient.

A limitation of the study was the small number of subjects. CWS was performed in an intubated and paraylzed state, which is different to prior CWS studies. However, LTx recipients are intubated during and immediately after the operation. While the porcine-pulmonary-model has many similarities to the human lung,^9^ the different anatomical distribution of intrathoracic organs, different shape of the rib cage and chest wall mechanics in pigs must be considered. The CWS changes in chest geometry have some similarities with RLD related chest cavity changes. However, a difference is that CWS did not alter the craniocaudal lung height. Lung perfusion and possible alterations via CWS were not assessed in this study-protocol. Furthermore, this porcine CWS-model does not include the physiological and immunological stress of LTx-operations.

In summary, we present the first description of a porcine-CWS-model as a model for the physiology of an oversized lung allograft. In future studies, the identification of a possible defined inflection-point for the physiological limits and characterization of the lung structure-function relationship and gas exchange efficiency when forcing the lung to operate at lower volumes via CWS could further inform acceptable ranges for size matching in LTx.^10^

## Financial support

This work was supported by a PILOT grant from the Institute for Clinical and Translational Science at the University of Iowa via the National Center for Advancing Translational Sciences Grant UL1-TR-000442 (M. Eberlein).

## CRediT authorship contribution statement

Eric Abston and Michael Eberlein contributed to Conceptualization, Performance of Experiments, Data curation, Formal analysis, writing and revising the manuscript.

## Conflicts of interest

The authors have no conflicts to declare.
